# Risk factors of active upper gastrointestinal bleeding in patients with COVID-19 infection and the effectiveness of PPI prophylaxis

**DOI:** 10.1186/s12876-022-02568-4

**Published:** 2022-11-17

**Authors:** Thaninee Prasoppokakorn, Pinit Kullavanijaya, Rapat Pittayanon

**Affiliations:** Division of Gastroenterology, Department of Medicine, Faculty of Medicine, Chulalongkorn University and King Chulalongkorn Memorial Hospital, Thai Red Cross Society, 1873 Rama IV Road, Pathumwan, Bangkok, 10330 Thailand

**Keywords:** Gastrointestinal bleeding, Gastrointestinal hemorrhage, Coronavirus-2019, COVID-2019, SARS-CoV-2

## Abstract

**Background:**

Gastrointestinal (GI) bleeding is one of the most impactful complications in patients hospitalized from COVID-19 infection. Limited study has focused on patients with upper GI bleeding (UGIB). This study aimed to identify the risk factors of patients who were hospitalized from COVID-19 infection and developed UGIB as well as the effectiveness of proton pump inhibitor (PPI) prophylaxis in those patients.

**Methods:**

This study was comprised of two phases. The first phase was the retrospective enrollment of patients who were admitted due to COVID-19 infection and developed UGIB between April and August 2021 to evaluate the associated factors of active UGIB. The second phase was a retrospective analysis after PPI prophylaxis protocol from September – October 2021 to assess the benefit of PPI use in those patients.

**Results:**

Of 6,373 patients hospitalized, 43 patients (0.7%) had evidence of UGIB. The majority were male 28 (65.1%) with a mean age of 69.1 ± 11.8 years. Twenty-four of 43 patients (55.8%) needed mechanical ventilation, 35 patients (81.4%) received systemic corticosteroids, and 10 patients (23.3%) were taking anticoagulants for venous thromboembolic prophylaxis. Seven of 43 patients (16%) had active UGIB. There was no significant difference in the number of patients taking antiplatelets, anticoagulants, or steroids and the severity of COVID-19 infection between the two groups. An emergency endoscopy or endoscopic hemostasis were performed in 6/7 (85.7%) patients. The multivariate logistic regression analysis revealed two significant factors associated with active UGIB including higher of Glasgow-Blatchford score (GBS) per point (OR = 7.89; 95%CI 1.03–72.87; *p* = 0.04) and an absence of PPI use (OR 4.29; 95%CI 1.04–19.51; *p* = 0.04). After prescribing PPI as a prophylaxis, there was a slightly lower incidence of UGIB (0.6% vs 0.7%) in addition to an absence of active UGIB (0% vs 16%).

**Conclusion:**

Our study demonstrated that the absence of PPI and higher GBS were significant risk factors for active UGIB which required therapeutic endoscopy in patients with COVID-19 infection. We suggest that short-term PPI prophylaxis should be prescribed in those patients once they need hospitalization regardless of the severity of COVID-19 infection to minimize the severity of UGIB.

## Introduction

The incidence of GI bleeding has been reported between 0.6–13% in patients hospitalized from COVID-19 infection [1, 2]. The mechanisms thought to cause GI bleeding include patient factors, viral factors eg. epithelial cells expressing the angiotensin 2-converting enzyme (ACE2), and indirectly from treatment-related consequences eg. systemic corticosteroids [3]. Previous data showed that patients with COVID-19 infection who developed either upper or lower GI bleeding during hospitalization had a significant higher mortality rate compared to those without GI bleeding [2].

A recent meta-analysis reported that between 37–100% of patients with COVID-19 infection who developed GI bleeding had taken anticoagulants and/or antiplatelet drugs [1]. However, there was insufficient data to test antiplatelet or anticoagulant as a predisposing factor of upper and lower GI bleeding in patients with COVID-19 infection [1]. Most recent studies assessed both upper and lower GI bleeding [1, 2]. To our knowledge, no study has evaluated the risk factors of active upper GI bleeding (UGIB) in patients with COVID-19 infection. Additionally, evidence supporting the use of proton pump inhibitors (PPI) which can prevent upper GI bleeding in high risk patients [4] is still lacking.

The primary purpose of our study was to evaluate the risk of active UGIB in patients with COVID-19 infection. The secondary purpose were 1) determining the etiology of UGIB; 2) rate of endoscopic hemostasis; 3) mortality rate of those patients and 4) the role of PPI prophylaxis.

## Methods

**Fig. 1:**
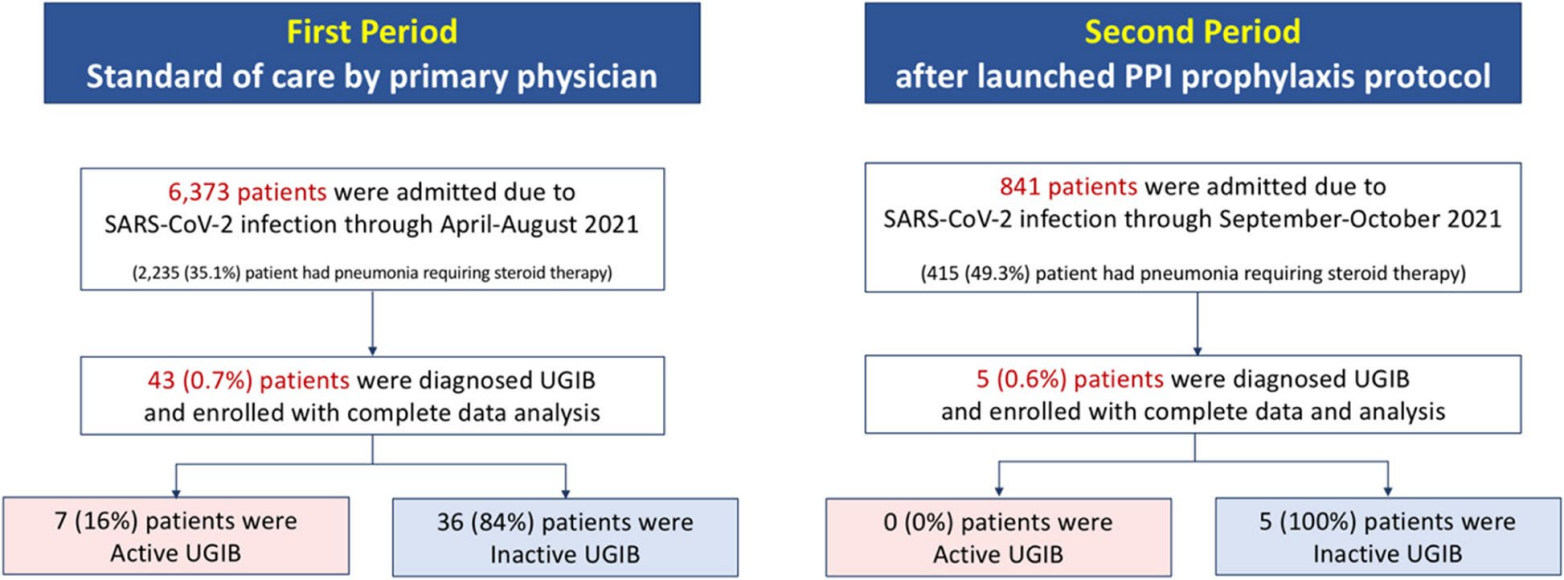
Flow diagram of two periods of patients enrollment before and after PPI prophylaxis protocol

This study was conducted at King Chulalongkorn Memorial Hospital, Bangkok, Thailand between April and October 2021. It was composed of 2 phases: 1) retrospective study, and 2) experimental study (Fig. 1). The outcome of this study was the hospitalized patients with COVID-19 infection who developed active UGIB.

### First phase: a retrospective study (April – August 2021)

We reviewed all charts of patients with COVID-19 infection who were admitted and developed UGIB during admission**.** The inclusion criteria were patients who were diagnosed with COVID-19 infection with evidence of GI bleeding. Confirmation of the diagnosis of SARS-CoV-2 infection was performed using a polymerase chain reaction from nasal and throat swabs. We recruited the patients by searching ICD-10 coding assistance under the following codes: GI bleeding (All K92 codes), hematemesis (K92.0), melena (K92.1), and hematochezia (K92.1, K75.31). In our hospital, ICD-10 coding was regularly documented by the primary physicians and routinely verified by the experienced senior physicians. Active UGIB was defined as having evidence of hematemesis, fresh blood from the nasogastric (NG) tube, or hematochezia combined with evidence of hemodynamic instability without other possible etiologies. An inactive UGIB was referred to as coffee ground emesis and/or melena. An emergency endoscopy was defined as having an endoscopy within 24 h of the first presentation of bleeding. If the patients were not undergone emergency endoscopy, they would be scheduled for diagnostic endoscopy within 90 days after the onset of the diagnosis of COVID-19 infection. Relevant data including past medical history, laboratory results, Glasgow-Blatchford score [5], severity of COVID-19 infection and treatment, type of endoscopy (diagnosis or therapy), clinical outcomes, length of hospital stay, and in-hospital mortality were collected. Patients under 18 years or had incomplete medical data were excluded.

### Second phase: an experimental study (September – October 2021)

We hypothesized that PPI might prevent active UGIB in COVID-19 patients. We launched the PPI prophylaxis protocol on 1^st^ September 2021 as a routine clinical practice for patients with high risk of GI bleeding. Patients with COVID-19 infection who need hospitalization for steroid or anticoagulant or both treatments would receive omeprazole 20 mg once daily (oral or intravenous route) regardless of endotracheal tube (ET-tube) intubation at the same date of admission. We followed patients for up to 2 months (September–October 2021) to determine the UGIB rate and retrospectively collected the data. The primary outcome was the incidence of UGIB after following this protocol. The secondary outcome was mortality rate.

The study protocol was approved by the Institutional Review Board of the Research Ethics Review Committee for Research Involving Human Research Participants, Health Sciences Group, Chulalongkorn University (IRB No.649/64). Data collection was initiated after IRB approval. This research was performed in accordance with the Declaration of Helsinki. This is a retrospective study, and signed informed consent was waived by the Ethics Committee. The analysis used anonymous clinical data after each patient agreed to treatment by written consent.

### Statistical analysis

All participants were assigned a numerical code for the study. Continuous variables were analyzed as mean ± standard deviation (SD) and compared using unpaired t-tests when data were normally distributed. Skewed variables were expressed as median (interquartile range; IQR) and compared using the Mann–Whitney U test. Categorical variables were compared using Fisher’s exact test or *X*^*2*^ test as appropriate. Binary logistic regression analysis, was performed to identify factors associated with active UGIB. The significant factors predicting active UGIB from univariate analysis and potential clinical factors that might relate to active UGIB (although they were not statistical significant from univariate analysis) were included in the multivariate model. The optimal cutoff score for detecting active UGIB was the value which provided the best sensitivity and specificity calculated using the area under the receiver operating characteristic (AUROC). Sample size was determined by estimating that 10 patients would develop active UGIB in order for the study to have adequate power to identify at least one significant factor. All statistical analyses were performed using the SPSS statistical analysis package version 22.0.0 (SPSS Inc., Chicago, Illinois, USA). A *p-*value of < 0.05 was considered statistically significant.

## Results

### First phase with retrospective study (standard of care) (Fig. 1)

A total of 6,373 patients were hospitalized due to COVID-19 infection. Of those, 43 patients (0.7%) were diagnosed with UGIB. There were 28 males (65.1%) and average age was 69.1 ± 11.8 years. The majority (31 patients, 72.1%) had multiple comorbidities including 31 patients (72.1%) with hypertension (HT), 27 (62.8%) with diabetes mellitus (DM), 13 (30.2%) with chronic kidney disease (CKD) or end-stage renal disease (ESRD), 9 (20.9%) with ischemic heart disease (IHD), 5 (11.6%) with cirrhosis, and 3 (6.8%) with old cerebrovascular accident (CVA). Twelve patients (27.9%) denied any comorbidity. The mean and median duration of COVID-19 infection before UGIB were 13.5 ± 12.5 and 8.0 (IQR 4.0, 22.0) days, respectively. There were 24 (55.8%) patients diagnosed with severe pneumonia requiring mechanical ventilation. Thirty-five (81.4%) patients received steroid therapy and 10 (23.3%) patients received anticoagulative agents including enoxaparin or heparin for venous thromboembolism (VTE) prophylaxis; 7 received enoxaparin and 3 received heparin (Table 1).

**Table 1:** Characteristics of COVID-19 infected patients with active UGIB compared to those with inactive UGIB (*n* = 43)

Variables	Total (*n* = 43)	Active UGIB (*n* = 7)	Inactive UGIB (*n* = 36)	*p*-value
Age (years)				
Mean ± SD	69.1 ± 11.8	67.0 ± 9.6	69.5 ± 12.3	0.466
Median (IQR)	68.0 (60.0,76.0)	67.0 (59.0,76.0)	68.5 (61.8,77.0)	
Sex Male, n(%)	28 (65.1)	5 (71.4)	23 (63.9)	0.737
**Comorbidities, n(%)**				
DM	27 (62.8)	3 (42.9)	24 (66.7)	0.213
HT	31 (72.1)	4 (57.2)	27 (75.0)	0.313
IHD	9 (20.9)	1 (14.3)	8 (22.2)	0.659
Old CVA	3 (7.0)	1 (14.3)	2 (5.6)	0.421
CKD/ESRD	13 (30.2)	0	13 (36.1)	0.082
Cirrhosis	5 (11.6)	2 (28.6)	3 (8.3)	0.218
**Presentations, n(%)**				
Hematemesis	5 (11.6)	5 (71.4)	0	< 0.001
Fresh blood from NG tube	1 (2.3)	1 (14.3)	0	
Coffee ground emesis	19 (44.2)	0	19 (52.8)	
Melena	17 (39.6)	0	17 (47.2)	
Hematochezia	1 (2.3)	1 (14.3)	0	
**COVID-19 infection details**				
Mechanical ventilator requirement, n(%)	24 (55.8)	5 (71.4)	19 (52.8)	0.338
COVID-19 infection date (day)				
Mean ± SD	13.5 ± 12.5	15.9 ± 15.5	13.1 ± 12.0	0.718
Median (IQR)	8.0 (4.0,22.0)	11.0 (1.0,30.0)	7.5 (4.0,21.5)	
**COVID-19 treatment, n(%)**				
Steroid	35 (81.4)	5 (71.4)	30 (83.3)	0.444
Enoxaparin/heparin	10 (23.3)	1 (14.3)	9 (25.0)	0.566
**Medications, n(%)**				
Antiplatelet	14 (32.6)	3 (42.9)	11 (30.6)	0.595
Anticoagulant	2 (4.7)	0	2 (5.6)	1.000
PPI	28 (65.1)	2 (28.6)	26 (72.2)	0.027
**Laboratories, mean ± SD**				
Hb baseline (g/dL)	11.5 ± 1.8	12.6 ± 1.7	11.3 ± 1.8	0.071
Hb (g/dL)	8.5 ± 2.0	7.5 ± 1.8	8.8 ± 2.0	0.136
Hb changing (g/dL)	3.0 ± 2.1	5.2 ± 1.8	2.6 ± 2.0	0.002
Hematocrit (%)	25.3 ± 6.1	22.5 ± 5.3	25.9 ± 6.1	0.187
INR	1.31 ± 0.37	1.41 ± 0.54	1.29 ± 0.33	0.411
PT	14.6 ± 3.9	15.6 ± 5.8	14.4 ± 3.5	0.444
PTT	27.3 ± 9.3	26.3 ± 9.5	27.6 ± 9.5	0.758
BUN (mg/dL)	57.7 ± 38.2	66.0 ± 26.6	56.1 ± 40.2	0.398
Creatinine (mg/dL)	3.05 ± 3.13	1.86 ± 0.57	3.29 ± 3.37	0.021
**Glasgow-Blatchford score,**				
Mean ± SD	10.7 + 4.0	15.4 ± 2.3	9.8 ± 3.6	< 0.001
Median (IQR)	11.0 (9.0,13.0)	14.0 (14.0,18.0)	10.5 (9.0,12.8)	
Glasgow-Blatchford score ≥ 14, n(%)	7 (16.3)	6 (85.7)	1 (2.8)	< 0.001

*UGIB* upper gastrointestinal bleeding, *BMI* body mass index, *DM* diabetes mellitus, *HT* hypertension, *IHD* ischemic heart disease, *CVA* Cerebrovascular accident, *CKD* Chronic kidney disease, *ESRD* end-stage renal disease, *PPI* proton-pump inhibitor, *Hb* hemoglobin, *WBC* white blood cell counts, *INR* international normalized ratio, *PT* prothrombin time, *PTT* partial thromboplastin time, *BUN* blood urea nitrogen, *SD* standard deviation, *IQR* interquartile range, *kg/m*^*2*^ kilogram per square meter, *g/dL* gram per deciliter, *cells/µL* cells per microliter, *mg/dL* milligram per deciliter

Among the 43 patients, seven patients had active UGIB (5 (11.6%) hematemesis, 1 (2.3%) hematochezia, and 1 (2.3%) fresh blood from NG tube). The remaining 36 patients had inactive UGIB (19 (44.2%) presented with coffee-ground emesis, 17 (39.6%) melena). The baseline characteristics comparing active and nonactive UGIB in the first phase of the cohort are shown in Table 1. There was no significant difference in the number of patients taking antiplatelets, anticoagulants, or steroids. Severity of COVID-19 infection (e.g. mechanical ventilator needed) between the two groups was also not statistically significant. The Glasgow-Blatchford score (GBS) in patients with active UGIB was significantly higher than those with inactive UGIB (median 14.0 vs 10.5; *p* < 0.001). Patients with current PPI use developed active UGIB less often than those without PPI (28.6% vs 72.2%; *p* = 0.027).

Among the 43 patients with UGIB, 17 patients (39.5%) underwent endoscopy, 11 had elective diagnostic endoscopy, and 6 received an emergency endoscopy. The emergency endoscopy was performed by experienced endoscopists, while the diagnostic endoscopies were completed by trainees under supervision. The emergency therapeutic endoscopies were performed in 6 of 7 (85.7%) patients. One patient with advanced stage bilateral breast cancer with multiple metastases was in moribund condition and denied esophagogastroduodenoscopy (EGD). She finally passed away from UGIB and advanced stage of cancer. Of the 6 patients who underwent endoscopies, there were 5 with high risk peptic ulcers and 1 with bleeding gastric lymphoma. All 6 patients underwent endoscopy hemostasis including 2 adrenaline injections plus hemoclip, 1 adrenaline injection plus bipolar coaptation, 1 adrenaline injection plus over-the-scope-clip, 1 adrenaline injection plus hemoclip and Hemospray®, and 1 bipolar coaptation plus over-the-scope clip. Three patients (42.9%) needed computed tomography angiography (CTA) followed by embolization after successful immediate endoscopic hemostasis. Two patients developed bleeding at 24 h after the index endoscopy and 1 patient underwent preemptive embolization to prevent recurrent bleeding. All 6 patients undergoing therapeutic EGD survived and were discharged from the hospital. Baseline characteristics and details of treatment and outcomes of all 7 patients with active UGIB are shown in Table 2.

**Table 2:** Characteristics of all COVID-19 infected patients with active UGIB (*n* = 7)

Patients	Age	Sex	Presentation	Comorbidities	COVID-19 date	Steroid/ Duration(day)	Anticoagulant/ Duration(day)	PPI prophylaxis	Delta Hb/ PRC(unit)	CTA/ Embolization	Endoscopy/ Finding	HP status	Management	Status/ GIH-related	LOS(day)
1	59	M	Hematemesis	Gastric DLBCL	1	Yes/1	No/-	No	6.5/13	Yes/Yes (hyperemia from a left gastric artery)	EGD/ BVV on gastric DLBCL involved hemi-circumferential upper body	Neg	Adrenaline injection, clipping, Hemospray® then gel foam embolization	Alive	33
2	67	F	Hematemesis	Bilateral breast cancers	19	Yes/13	Yes/12	No	5.2/0 (BSC)	No/No	Not done	NA	NA	Expired/ yes	2
3	60	M	Hematemesis	Alcoholic cirrhosis, ACLF	1	No/-	No/-	No	4.6/1	No/No	EGD/ 2.5-cm NBVV antral ulcer	Neg	Adrenaline injection, bipolar coaptation	Alive	28
4	55	M	Hematochezia	Old CVA HT DLP	11	Yes/11	No/-	No	3.4/2	No/No	EGD/ 1-cm NBVV antral ulcer	Pos	Bipolar coaptation then over-the-scope-clip	Alive	9
5	71	M	Hematemesis	DM HT DLP	30	Yes/5	No/-	Yes	6.9/10	No/No	EGD/ 0.5-cm BVV duodenal bulb	Neg	Adrenaline injection then over-the-scope-clip* duodenal perforation required surgical wedge resection	Alive	37
6	76	M	Hematemesis	HBV cirrhosis decompensation	7	No/-	No/-	Yes	2.5/6	Yes/Yes (active extravasation at duodenal wall via duodenal branch GDA)	EGD/ 1-cm BVV duodenal bulb	Neg	Adrenaline injection, clipping, Glue embolization	Expired/ yes	14
7	81	F	Fresh blood from NG tube	IHD DM HT DLP	42	Yes/15	No/-	No	7.1/4	Yes/Yes (no contrast extravasation, no pseudoaneurysm)	EGD/ 0.8-cm NBVV on 5-cm ulcer at duodenal bulb	Pos	Adrenaline injection, clipping then preemptive coil embolization	Alive	6

*UGIB* upper gastrointestinal bleeding, *DLBCL* diffuse large B-cell lymphoma, *ACLF* acute-on-chronic liver failure, *CVA* cerebrovascular accident, *HT* hypertension, *DLP* dyslipidemia, *DM* diabetes mellitus, *HBV* hepatitis B virus, *IHD* ischemic heart disease, *CKD* chronic kidney disease, *HCC* hepatocellular carcinoma, *DVT* deep vein thrombosis, *PE* pulmonary embolism, *NG* nasogastric, *PPI* proton-pump inhibitor, *Hb* hemoglobin, *PRC* pack red cells, *CTA* computed tomography angiography, *GDA* gastroduodenal artery, *EGD* esophagogastroduodenoscopy, *NBVV* non-bleeding visible vessel, *BVV* bleeding visible vessel, *HP* Helicobacter pylori *Pos* positive, *Neg* negative, *LOS* length of stay, *BSC* best supportive care, *NA* not applicable

**Fig. 2:**
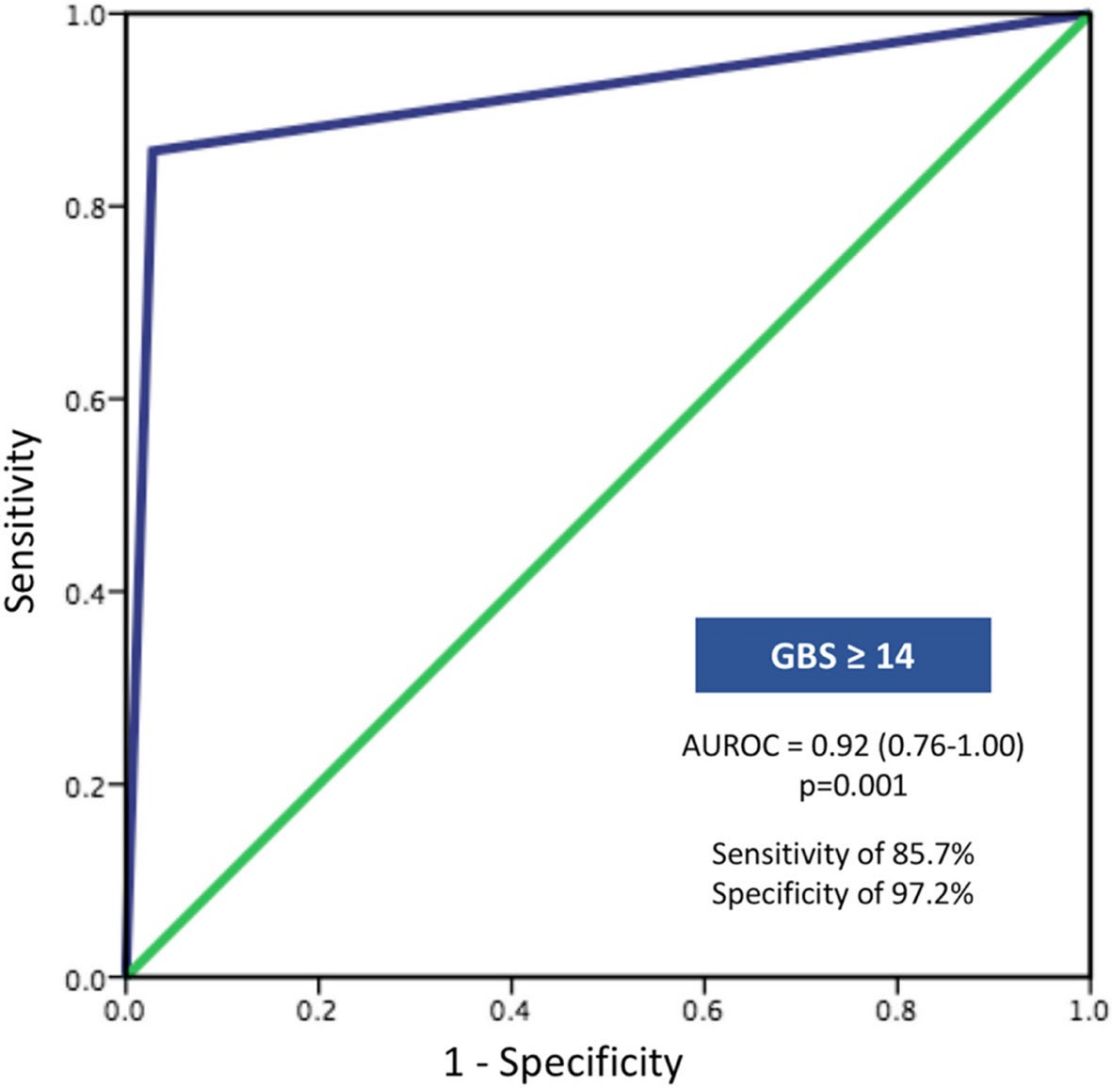
The AUROC curve of Glasgow-Blatchford score more than 14 for active UGIB detection

The outcomes of patients with COVID-19 infection who developed UGIB are shown in Table 3. Patients with active UGIB required a greater amount of pack red cell (PRC) transfusion compared to those with inactive UGIB (5.1 ± 4.8 vs 1.8 ± 1.9 units; *p* = 0.003) and had more CTA with hemostatic embolization compared to inactive UGIB (42.9% vs 0.0%; *p* < 0.001)**.** The overall mortality rate of patients with UGIB was 17 of 43 (39.5%). No statistically significant difference in mortality rate between patients with active and inactive UGIB was observed (28.6% vs. 41.7%; *p* = 0.551). The univariate analysis consecutive multivariate logistic regression analysis showed two significant factors associated with active UGIB including higher of GBS per point provided OR = 7.89; 95%CI 1.03–72.87 (*p* = 0.04), and the absence of PPI use provided OR = 4.29; 95%CI 1.04–19.51 (*p* = 0.04) (Table 4). The diagnostic performance of GBS 14 or more provided sensitivity and specificity of 85.7% and 97.2%, respectively, PPV and NPV of 85.7% and 97.2%, respectively, and produced an AUROC of 0.92 (95%CI 0.76–1.00, *p* = 0.001) for active UGIB detection (Fig. 2). Using the GBS cut point 14 or more, we were able to discriminate between patients needing therapeutic EGD and those who did not need EGD with a sensitivity and specificity of 85.7 and 97.2%, respectively.

**Table 3:** Outcomes of COVID-19 infected patients with active UGIB compared to those with inactive UGIB (*n* = 43)

Variables	Total (*n* = 43)	Active UGIB (*n* = 7)	Inactive UGIB (*n* = 36)	*p*-value
**Treatment**				
PRC (units)				
Mean ± SD	2.3 ± 2.8	5.1 ± 4.8	1.8 ± 1.9	0.003
Median (IQR)	1.0 (0.0,4.0)	4.0 (1.0,10.0)	1.0 (0.0,3.0)	
CTA with embolization, n(%)	3 (7.0)	3 (42.9)	0 (0.0)	< 0.001
**Endoscopic findings, n(%)**	*n* = 17	*n* = 6	*n* = 11	0.328
Gastroduodenal ulcer	11 (64.7)	5 (83.3)	6 (54.5)	
Gastritis	3 (17.6)	0	3 (27.3)	
Esophagitis	1 (5.9)	0	1 (9.1)	
Gastric lymphoma	1 (5.9)	1 (16.7)	0	
Normal (EGD, colonoscopy)	1 (5.9)	0	1 (9.1)	
**HP positive, n(%)**	3/17 (17.6)	2/6 (33.3)	1/11 (9.1)	< 0.001
**Outcomes**				
Expired, n(%)	17 (39.5)	2 (28.6)	15 (41.7)	0.551
GIB-related death, n(%)	1 (2.3)	1 (14.3)	0	< 0.001
LOS (days)				
Mean ± SD	31.7 ± 30.6	18.4 ± 14.0	34.2 ± 32.4	0.215
Median (IQR)	25.0 (14.0,37.0)	14.0 (6.0,33.0)	25.0 (15.0,45.0)	

*UGIB* upper gastrointestinal bleeding, *PRC* pack red cells, *CTA* computed tomography angiography, *HP Helicobacter pylori*, *LOS* length of stay. *SD* standard deviation, *IQR* interquartile range, *kg/m*^*2*^ kilogram per square meter

**Table 4:** Univariate and multivariate logistic regression analysis of factors associated with active UGIB

Variables	Univariate		Multivariate	
	**OR (95%CI)**	***p*-value**	**OR (95%CI)**	***p*-value**
Cirrhosis	3.30 (0.47–22.98)	0.22	2.65 (0.26–27.28)	0.41
Mechanical ventilator requirement	2.37 (0.41–13.79)	0.34	4.61 (0.53–40.30)	0.17
Steroid use	2.07 (0.32–13.25)	0.44	2.05 (0.21–20.38)	0.54
Prophylactic enoxaparin/ heparin use for COVID-19 treatment	1.93 (0.20–18.23)	0.57	1.02 (0.06–16.71)	0.89
Current antiplatelet use^a^	1.56 (0.30–8.12)	0.60	3.51 (0.33–37.55)	0.30
Current anticoagulant use^a^	0.83 (0.09–7.54)	1.00	NA	1.00
**No PPI prophylaxis**	**4.41 (1.10–20.33)**	**0.03**	**4.29 (1.04–19.51)**	**0.04**
**GBS (every1 point)**	**5.89 (1.15–30.05)**	**0.03**	**7.98 (1.03–72.87)**	**0.04**

*PPI* proton-pump inhibitor, *GBS* Glasgow-Blatchford score**,** *OR* odd ratio, *CI* confidence interval

^a^Received treatment for underlying disease prior to admission

### Second phase with retrospective study after PPI prophylaxis protocol (Fig. 1)

A total of 841 patients were hospitalized due to COVID-19 infection during the 2-month study period (September–October 2021). All patients were prescribed standard dose of PPI prophylaxis during admission. Five of these patients (0.6%) were diagnosed with UGIB. Of these 5 patients, there were 2 (40.0%) males and mean age of 74.2 ± 11.5 years. Three patients (60.0%) did not have any comorbidities, while 2 patients (40.0%) had HT and 1 (20.0%) had DM, IHD, and cirrhosis. All patients with inactive UGIB presented with coffee-ground emesis (5 patients, 100%) and did not undergo endoscopy due to low risk of UGIB. They were prescribed high dose PPI (twice daily) and discharged from the hospital. No bleeding had occurred at 1 month follow-up. PPI was prescribed for approximately two weeks or less and stop before patient’s discharge. There has been no report of PPI-related side effects, particularly increasing respiratory infection or gastrointestinal infection.

## Discussion

Our present study demonstrated an overall incidence of UGIB in patients with SARS-CoV-2 infection who needed hospitalization of 0.7% (44/6,373 patients). This number was slightly decreased to 0.6% (5/841) after implementing a standard dose of PPI prophylaxis. Importantly, the percentage of patients presenting with active UGIB was reduced from 16 to 0% after prescribing PPI prophylaxis to patients taking steroids and/or anticoagulants. This information supported our first phase data that the absence of PPI prophylaxis was the significant factor associated with active UGIB. In addition, our first phase data showed 26% absolute risk reduction (ARR) when using PPI to prevent active UGIB in patients who admitted with COVID-19 and provided number needed to treat (NNT) of 4. Previous studies had combined data from UGIB and LGIB [2, 6]. To our knowledge, this is the first study that focuses solely on UGIB in COVID-19 patients.

A large retrospective cohort in the United States demonstrated that using antiplatelet or anticoagulant agents was not a risk factor for GI bleeding [2]. Our study supported these findings on the prior use of antiplatelet/anticoagulant and risk of GI bleeding. Patients recently receiving anticoagulant for VTE prophylaxis for COVID-19 infection were also not found to be a risk factor for active UGIB.

The mortality of COVID-19 infected patients with GI bleeding has been previously reported inconclusively [1, 2, 6]. A propensity score-matched case–control study reported that the occurrence of GI bleeding during hospitalization is associated with a significantly increased mortality rate compared to those without GI bleeding with OR 1.58 (95% CI 1.06–2.34, *p* = 0.02) [2]. A second matched case–control study reported that in-hospital mortality rates were similar between patients with and without GI bleeding [6]. Recently, a meta-analysis study reported no significant association between GI bleeding and overall mortality rate [1]. In our cohort study, we demonstrated no difference in the overall mortality rate of patients with active UGIB group compared to those with inactive UGIB group.

The current guidelines have suggested using PPI as co-therapy in patients requiring anticoagulants only if they had a history of peptic ulcer bleeding [7]. There was no recommendation for prescribing PPI when patients were taking only anticoagulants or corticosteroids without any risk of UGIB. However, we know that patients taking anticoagulant have a risk of bleeding [8]. Two systematic reviews revealed that corticosteroids increased the incidence of GI bleeding, especially in critically ill patients [9, 10]. Thus, we proposed the short-term PPI prophylaxis protocol for patients with COVID-19 infection who received anticoagulants and/or corticosteroids and found it could prevent active UGIB during hospitalization. This may support the future societal guideline on this issue.

This study reported a need for therapeutic endoscopy of 14% for overall GI bleeding and 100% in patients presenting with the clinical of active UGIB. Our data did not differ from previous studies which reported an overall therapeutic endoscopic hemostasis rate between 6–48% [2, 11, 12]. However, no previous study evaluated the need for therapeutic endoscopy in patients with active GI bleeding.

Regarding the Glasgow-Blatchford score (GBS) which is a screening method for determining whether patient with an acute UGIB will likely require endoscopic hemostatic procedure [5]. The performance of the GBS to predict the need for therapeutic intervention and blood transfusion has been widely validated in the previous studies [13, 14]. According to guidelines for UGIB management, it is recommended to perform EGD within the first 24 h after presentation in patients with a GBS of more than 1 [4, 15, 16]. Although the patients in this study had inactive UGIB, they had a median GBS at 10.5. If we had followed the guidelines, we would have had to perform EGD in those patients. This study demonstrated that the threshold for early endoscopy within 24 h in patients with COVID-19 and UGIB should be revised. Those patients could be given more conservative treatment with high-dose PPI and supportive treatment.

A systematic review from the United States showed that 108 of 123 (87.8%) patients with overall GI bleeding were managed conservatively with PPIs, somatostatin analogs, vasopressin analogs, and intravenous fluid resuscitation [17]. Experts from Italy and United States suggest postponing non-urgent endoscopy in those patients [18, 19]. Performing EGD in patients with COVID-19 infection consumes many resources and is costly. The procedure has a high risk of viral shedding which requires a multidisciplinary team, personal protective equipment, and an appropriate negative pressure room. Our study proposed a GBS threshold of 14 or more supported by an excellent AUROC for selecting patients most likely to benefit from urgent EGD.

Our study had certain limitations. First, the number of patients with active upper GI bleeding is limited. Significant testing on mortality rates was likely impacted by the small sample size. Despite the limited sample size, this study was able to demonstrate two significant factors in the multivariable analysis. Second, since our study was a retrospectively design, there were definitely biases which might affect the outcomes, for instance, the incomplete data of tobacco use, alcohol consumption and *Helicobacter pylori* status. Finally, this study recruited small proportion of patients having an active UGIB in the first phase of study, and this can affect the result of the multivariate regression analysis. This limitation was highlighted by the large 95% CI obtained in the analysis. Nevertheless, this study demonstrated the data from a real-life practice on the emerging disease. A future prospective study on the benefits of PPI for UGIB prevention in patients with emerging viral disease e.g. COVID-19 should be further explored. After incorporating the results of this study with 0.05 alpha and 0.20 beta, the sample size of PPI and non-PPI group in the future prospective randomization study was 36 each.

## Conclusion

This study demonstrated that absence of PPI use and high GBS of 14 or more were significant risk factors for active UGIB requiring therapeutic endoscopy in patients with COVID-19 infection. Results suggest that short-term PPI prophylaxis may be considered in hospitalized patients to minimize the severity of UGIB. A prospective research on the benefit and risk of PPI for UGIB prevention in patients with emerging respiratory tract infection should be further explored.

## Data Availability

The datasets generated and analyzed during the current study are not publicly available but are available from the corresponding author on reasonable request.

## Abbreviations

GI: Gastrointestinal
UGIB: Upper gastrointestinal bleeding
PPI: Proton pump inhibitor
GBS: Glasgow-Blatchford score
ACE2: Angiotensin 2-converting enzyme
NG: Nasogastric
ET: Endotracheal
SD: Standard deviation
IQR: Interquartile range
AUROC: Area under the receiver operating characteristic
OR: Odds ratio
HT: Hypertension
DM: Diabetes mellitus
CKD: Chronic kidney disease
ESRD: End-stage renal disease
IHD: Ischemic heart disease
CVA: Cerebrovascular accident
VTE: Venous thromboembolism
CTA: Computed tomography angiography
EGD: Esophagogastroduodenoscopy
PRC: Pack red cell
